# Fault Detection of Aircraft System with Random Forest Algorithm and Similarity Measure

**DOI:** 10.1155/2014/727359

**Published:** 2014-06-26

**Authors:** Sanghyuk Lee, Wookje Park, Sikhang Jung

**Affiliations:** ^1^Department of Electrical and Electronic Engineering, Xi'an Jiaotong-Liverpool University, Suzhou 215123, China; ^2^Aviation Technical Division, Aviation Safety Technology Center, 557 Yongyu-ro, Jung-gu, Incheon 400-420, Republic of Korea; ^3^Department of Aerospace, Automobile & Mechanical Engineering, Chung Cheong University, 38 Wolgok-Gil, Gangnae-Myeon, Cheongwon-Gun, Chungcheongbuk-Do 363-792, Republic of Korea

## Abstract

Research on fault detection algorithm was developed with the similarity measure and random forest algorithm. The organized algorithm was applied to unmanned aircraft vehicle (UAV) that was readied by us. Similarity measure was designed by the help of distance information, and its usefulness was also verified by proof. Fault decision was carried out by calculation of weighted similarity measure. Twelve available coefficients among healthy and faulty status data group were used to determine the decision. Similarity measure weighting was done and obtained through random forest algorithm (RFA); RF provides data priority. In order to get a fast response of decision, a limited number of coefficients was also considered. Relation of detection rate and amount of feature data were analyzed and illustrated. By repeated trial of similarity calculation, useful data amount was obtained.

## 1. Introduction

Fault-tolerance and adaptation of aircraft system with actual faults/healthy data have been studied. In order to process the adaptation of the pilot or the flight control system under abnormal condition, critical mission or return to a safe region should be followed with clear and right decision [1]. First of all, data for fault decision should be available; data acquisition system is required to take data accurately and quickly for the guarantee of precise fault decision. A lightweight fault detection model of DURUMI-II, which is an unmanned aerial vehicle (UAV), was considered and fault decision process was carried out with random forests algorithm (RFA) [2]. RFA is a state-of-the-art classification algorithm and has shown high classification accuracy. Additionally, RFA generates priority for each feature. The proposed approach enables one to figure out stable, important features with high detection rates. As a result, parameter optimization and feature selection were performed to make guaranteed high detection rates.

In order to get fault detection result, discrimination measure has to be considered. Similarity measure [3–6] represents the degree of similarity between comparable data; it has also been done by numerous works [1, 2, 7–9]. Similarity measures between two vague datasets mean that it roughly depends on inverse value of distance. Therefore, similarity can be considered as common information between two data distributions; hence, the obtained similarity measure was based upon the distance measure. Hence, the computation of similarity between two fuzzy sets could be followed with the obtained similarity measure.

12 cases operating data are used to design discriminating measure for healthy/faults condition. Hence, it is represented as multivariate dataset; it is also understood as multidimensional data. Each component has different importance to determine either faulty or healthy. To get more accurate decision, it is needed to consider weighting factor to each 12 coefficients. Depending on expert's opinion could be a strong candidate to solve the problem. It seems, like the heuristic approach, results depend on individual. It means that results cannot guarantee the consistency. RFA provides important values with respect to each feature. It means that more rationale can be obtained through RFA.

In the previous research [1, 10, 11], fault detection and isolation (FDI) operation were accompanied by the fault-tolerant control system to control process failure. With the assumption of the controllability and the trimmability of the UAV at postfailure conditions, it means that the aircraft can keep on flying with the help of 6 flight control computer even the control surface stuck happen. It makes restructure and reconfigure controllers according to the grade of system failure. To get a decision or analysis for faults, statistical information of elevator deflection, aileron deflection, and others has been analyzed.

By applying RFA to multivariate similarity measure, different weighting factors were considered. Detection results showed good performance with actual data. Specially, when data was placed within overlapping region, RFA showed satisfactory performance compared to unitary weighting case [7, 8]. In the next chapter, RFA was introduced. Normal operation and longitudinal faulty operation were performed, and operation data were obtained. Considered airplane model and state equation were also illustrated in Chapter 3. RFA procedure was introduced in Chapter 4, and the importance value was obtained through RFA. Computation result was compared with similarity measure calculation. Similarity was also weighted with importance variable. Finally, conclusions are derived.

## 2. Random Forests Algorithm

A lot of interest in ensemble learning algorithm generated many results about classifier and regression by way of boosting and bagging [2, 7, 8, 12–14]. Random forests algorithm was proposed by Breiman [2], the method is categorized under “ensemble learning” method, and it adds an additional layer of randomness to bagging. Applying ensembles of trees can achieve important gains in classification and regression accuracy and each tree in the ensemble is developed according to the random parameter. Applying an injection of randomness, each of these trees is generated. Dietterich proposed random split selection approach, where the split was selected randomly from the *K* best splits at each node [8].

The common element in all RFA procedures is multiple trees. Random vector Θ_*k*_ is generated from past random vectors Θ_1_,…, Θ_*k*−1_, each has the same distribution independently. By the derivation of Breiman, random vector Θ is generated as the counts in *N* boxes. It is resulting from *N* darts thrown at random at the boxes, where *N* is the number of examples in the training set. Then, Θ consists of a number of independent random integers between 1 and *K*. A tree is also grown with the training set and Θ_*k*_ and resulting in a classifier *h*(**x**, Θ_*k*_) where **x** is an input vector [2]. With the definition of Breiman, RFA is defined in Definition 1.

**Definition 1 (see [2]).**

A random forest is a classifier consisting of a collection of tree-structured classifiers {*h*(**x**, Θ_*k*_), *k* = 1, …} where the {Θ_*k*_} are independent identically distributed random vectors and each tree casts a unit vote for the most popular class at input **x**.

With the property of Definition 1, two missions are needed to design RFA, one is a tree structure and the other is the number of trees. Tree structure has no rule for the design; it depends on computational requirement and designer himself/herself. The number of trees should be decided through heuristic consideration such as by using trial and error. Well-known tree algorithm is considered in reference [12].

**Figure 1:**
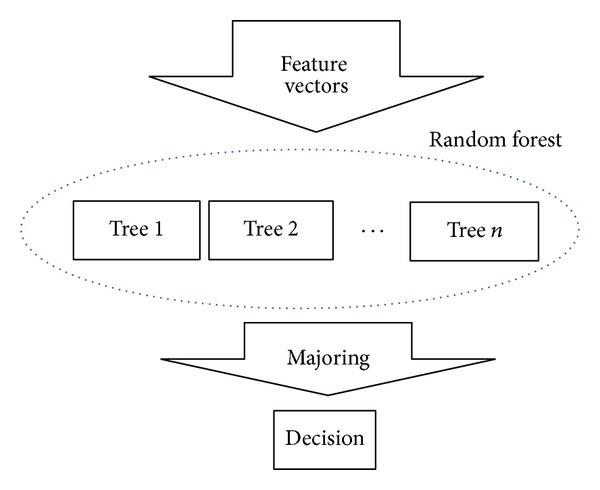
Structure of RFA.

Total structure of RFA is shown in Figure 1. Trees are repeated *n* times. As in reference [12], RFA has several advantages and disadvantages. It is one of the effective learning algorithms, because it is convenient to process large database, and possible to handle many input variables. However, it often shows overfit for some datasets, and not easy to interpret, and others. Even some uncomfortable RFA results provide useful information such as variable importance, because decision result could be obtained through probability.

Majoring could be obtained by averaging each class result. By overlapping the results of each tree decision, it also provides ensemble mean of each decision result. Effect of deleting uncertainty was done by averaging node outputs. Furthermore, if feature vectors have wide characteristics, then it is obviously guarantee ergodicity.

## 3. Model for Similarity Measure

### 3.1. Airplane Model

Consider the aircraft system of combining with longitudinal mode and lateral-directional mode. Then, the state space equation is as follows [1, 10, 11, 15–17]:

(1) $$\begin{array}{c} \dot{x}\left(t\right)=Ax\left(t\right)+Bu\left(t\right),\\ y\left(t\right)=Cx\left(t\right)+Du\left(t\right), \end{array}$$

where **x** and **u** denote state vector and elevator control input variable, respectively. Output vector **y** is identified by **x** itself [11, 15–17]. Consider

(2) $$\begin{array}{c} x\;=\;{\left[\alpha \;\;u\;\;q\;\;\theta \;\;\beta \;\;p\;\;\gamma \;\;\phi \right]}^{T},\\ u\;=\;\delta_{e}, \end{array}$$

where **u**  =  *δ*_*e*_ is elevator control input. In the state vector, *p*, *q*, and *γ* are the angular velocities and *α* and *β* are the angle of attack and the sideslip angle. Finally, *ϕ* and *θ* represent the roll and pitch angle, respectively.

**Figure 2:**
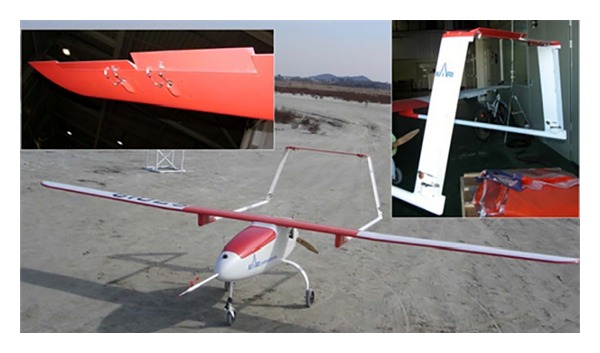
The UAV configuration.

In Figure 2, an experimental model consisted of one elevator, one rudder, and two ailerons. In order to get two types of data, normal and fault, two times of data acquisitions were carried out. It is notified that one or two of elevator, aileron, and rudder were broken intentionally. It was made after taking normal operating data. In order to simulate our fault detection procedure, the experimental model also has been equipped with one-piece elevator. It was separated into two—one is normal and the other is faulty. Hence, it was difficult to know whether it was faulty or not. Therefore, control surface has been added at the other vertical stabilizer. Considered UAV is illustrated in Figure 2; fault was applied artificially. Two different flight tests were carried out for normal and faulty operations separately [1, 10, 11].

In order to carry out the experiment, intentional damages were applied to the right elevator, the left rudder, and the left aileron stuck and the combination of this surface stuck was considered. Without the uncontrollability and the untrimmability of the aircraft at postfailure conditions, the flight test was scheduled. For left rudder only and right elevator with the left rudder stuck cases were considered. Stuck angles of the first case (left rudder only) were considered from −10°, −5°, 0°, and +5°, to +10°. Same stuck angles of the second case (right elevator with the left rudder stuck) were also considered.

In the first flight test, the control input for the real-time parameter estimation was applied with the knob and switching method, and the flight data was obtained from the exciting dynamics of UAV operation with the mentioned method [1, 10, 14]. However, as it was pointed out in the result, result showed that the applied time interval was slightly inaccurate. So, in the second flight test, for the purpose of constant control realization and the time interval, the control input device was developed to use an RF modem and a R/C transmitter [1, 10, 11].

### 3.2. Parameters of Longitudinal Mode

In order to control UAV during the occurrence of surface stuck and combination stuck, the aircraft should possess the controllability and the trimmability under the postfailure conditions. Because flight test procedure contains the ability to recover to the normal state from the fault state [1, 10, 11], Table 1 shows that the experimental results of UAV (DURUMI-II) show the trim value of available primary control surface at postfailure conditions. Blank was considered as the uncontrollability and the untrimmability cases.

Research on longitudinal mode fault detection was carried out in the previous research [10]. In order to obtain the failure status of the elevator, analyses of *C*_*m*_*δe*__, *C*_*m*_*α*__, and *C*_*L*_*α*__ were essential to obtain information on the aircraft performance characteristics. In the research, instead of statistical information such as mean and variance similarity measure was proposed to overcome the ambiguity of big standard deviation. Because it invokes scattering results, the analysis results of the stability and controllability derivatives are not clear.

## 4. Numerical Results and Their Analysis

Fault detection algorithm was proposed in this chapter. Total of 89 data were considered, 38 normal and 51 fault. Twelve features were also included within dataset.

### 4.1. Random Forest with Numerical Interpolation

Considering RFA, the number of variables in the random subset at each node (*m*_try_) and the number of trees in the forest (*n*_tree_) are needed. In order to get the best classification rate (correction decision rate), determination of optimal number of two parameters is required.

Requirements to decide operation condition are considered as follows.

*Requirement 1*. Parameters optimization is conducted to guarantee high detection rates. 

*Requirement 2*. Fault detection model is built using RFA with all features. The RFA generates variable importance values in numerical form. 

*Requirement 3*. Rank whole features and eliminate the irrelevant features. 

*Requirement 4*. Rebuild a fault detection model with only *k* selected features. 

*Requirement 5*. Evaluate the rebuilt detection model. If the detection rates and error rates satisfy requirements, terminate the computation. Otherwise, iterate with less number of features.

To evaluate the feasibility of our approach, longitudinal experiments on the flight test data of Table 3 are considered [18].

**Figure 3:**
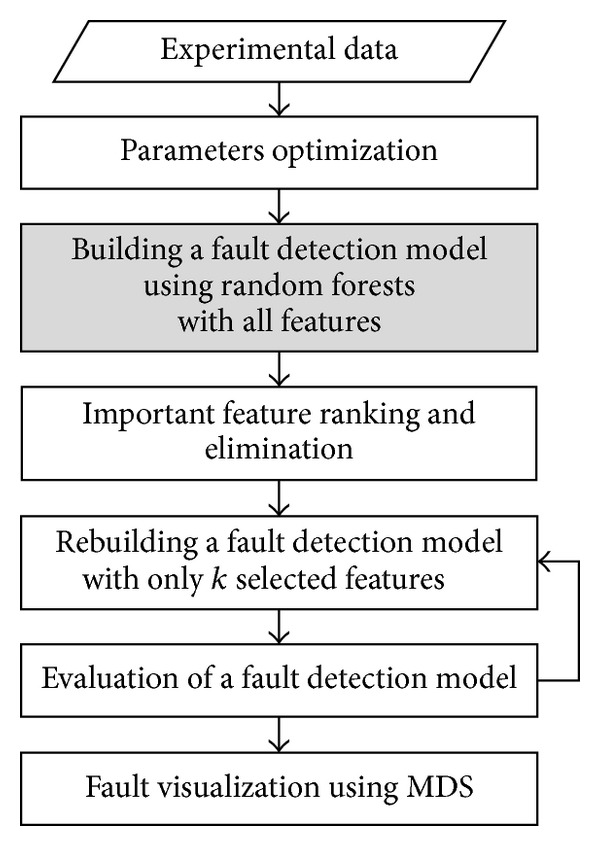
Overall diagram of proposed sequences.

In Figure 3 sequence, the highest detection rate was satisfied when *m*_try_ was used only with 2 features. For *n*_tree_, there is no specific function that figures out the optimal value as *m*_try_. Thus, the optimal value of *n*_tree_ was considered by choosing the *n*_tree_ value as high and stable detection rates.

As results of experiments, two optimized parameter values were considered when *m*_try_ = 2 and *n*_tree_ = 260. With these two parameters, feature selection of the flight test data has been carried out by employing the feature selection algorithm supported by RFA.

RFA provides the variable importance of each feature; its results are illustrated in Table 2. The proposed approach shows reasonable context information by their important feature. Here, the meaning of *C*_*m*_*δe*__, *C*_*m*_*α*__, and *C*_*L*_*α*__ and other parameters are expressed in reference [10, 11]. By the results of Table 1, the pitching moment coefficient with changes in the elevator deflection *C*_*m*_*δe*__ shows bigger difference than the other parameters. However, to get more reliable data, detection ratio versus number of parameters was also carried out.

**Figure 4:**
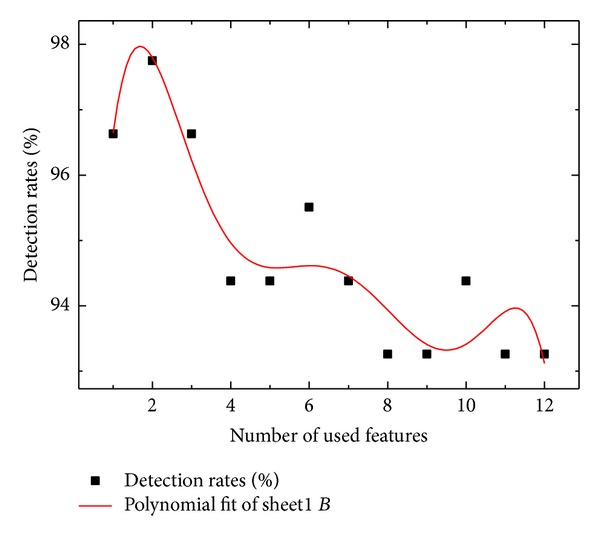
Detection rate with respect to number of features.

**Figure 5:**
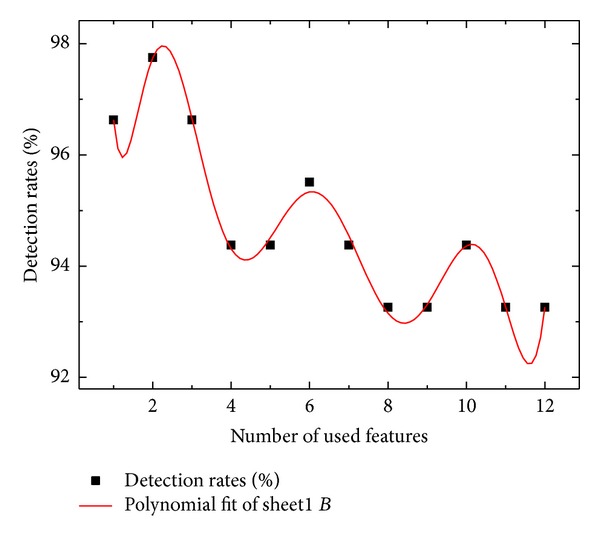
Detection rate with respect to number of features.

This approach shows that the feature selection should be important to decide decision performance because the number of features determines detection rate. With combinations of the highest importance variable, decision rate was obtained. Results were illustrated in Figures 4 and 5. By numerical conclusion, the highest decision rate, 97.75%, was obtained when the highest two features were used.

Interpolating the data with sixth order polynomial was obtained as

(3) $$\begin{array}{c} y=a+b_{1}x+b_{2}x^{2}+\cdots +b_{6}x^{6}. \end{array}$$

Intersecting value and coefficients *b*_1_ to *b*_6_ are illustrated in Table 3.

By differentiating (3), three maxima satisfy 1.67, 6.00, and 11.22.

These values mean number of features. Hence, it is definite that two features selection guarantees highest detection rate as in Figure 4. Similarly, 9th order polynomial interpolation was obtained as follows:

(4) $$\begin{array}{c} y=a+b_{1}x+b_{2}x^{2}+\cdots +b_{9}x^{9}. \end{array}$$

Next, three maxima are also obtained as 2.11, 6.11, and 10.11 (Table 4).

It shows the same result with 6th order polynomial interpolation.

**Figure 6:**
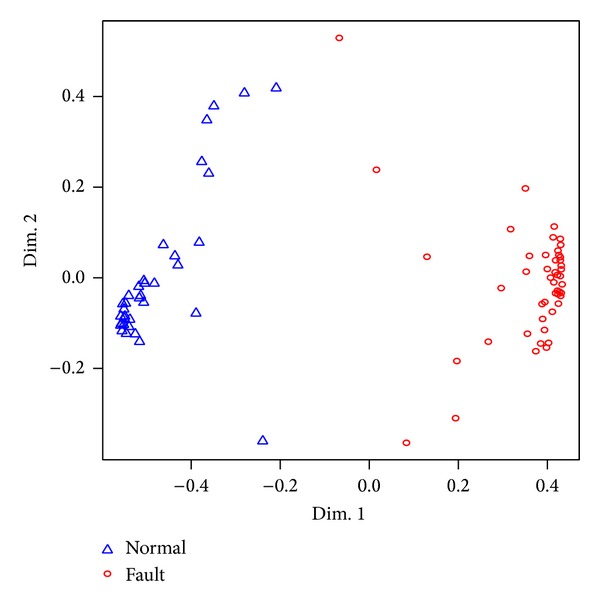
MDS plots of normal and fault patterns.

By considering multidimensional scaling (MDS), it provides a method for discovering “hidden” structures in multidimensional data. MDS is designed by considering similarity measure and mapped on a lower dimensional spatial representation [12, 13]. With coefficients *C*_*m*_*δe*__, *C*_*m*_*α*__, and *C*_*L*_*α*__, normal/fault patterns are implemented by multidimensional scaling (MDS) methodology in Figure 6 [7]. It is obtained with open source *R*-project [12].

Above results provide two parameters, *C*_*m*_*δe*__ and *C*_*m*_*α*__, which are enough and most efficient to decide whether it is faulty or not. Now, normal and fault patterns are illustrated via MDS. Normal and fault patterns are shown via blue triangles and red circles in Figure 6. This indicates that the fault monitoring and flight control system organization can be feasible by visualization, without expert's knowledge.

### 4.2. Comparison with Similarity Measure Results

Similarity measure is designed through using the definition of Liu [19]. Following similarity measure will be used as the calculation of the degree of similarity between normal and fault operating conditions. Proposed similarity measure has strong point by the point of computation in comparison to the result of the literatures [3–6, 20–22]. They required at least 2*n* comparisons and 2*n* additions by the formations of

(5) s(A,B)=1−d(A,A∩B)−d(B,A∩B),s(A,B)=2−d((A∩B),[1]X)−d((A∪B),[0]X).

**Theorem 2 .**

For any sets *A*, *B* ∈ *F*(*X*),

(6) $$\begin{array}{c} s\left(A,B\right)=1-d\left(\mu_{A}\left(x\right),\mu_{B}\left(x\right)\right) \end{array}$$

is the similarity measure between set *A* and set *B*, where *d* satisfies Hamming distance measure.

**Proof**

Commutative property of (S1) is easy to prove; it is clear from (6) itself. To show (S2);

(7) s(D,DC)=1−d(μD(x),μDC(x))=1−1=0

is obtained. Because *μ*_*D*_(*x*) and *μ*_*D*^*C*^_(*x*) are complements, difference *d*(*μ*_*D*_(*x*), *μ*_*D*^*C*^_(*x*)) always satisfies one. (S3) is rather easy to prove:

(8) $$\begin{array}{c} s\left(C,C\right)=1-d\left(\mu_{C}\left(x\right),\mu_{C}\left(x\right)\right)=1. \end{array}$$

From above statement, it is rational that *s*(*C*, *C*) satisfies maximal value. Finally, triangular equality is obvious by the definition of Liu [19]; hence (S4), is also satisfied.

**Figure 7:**
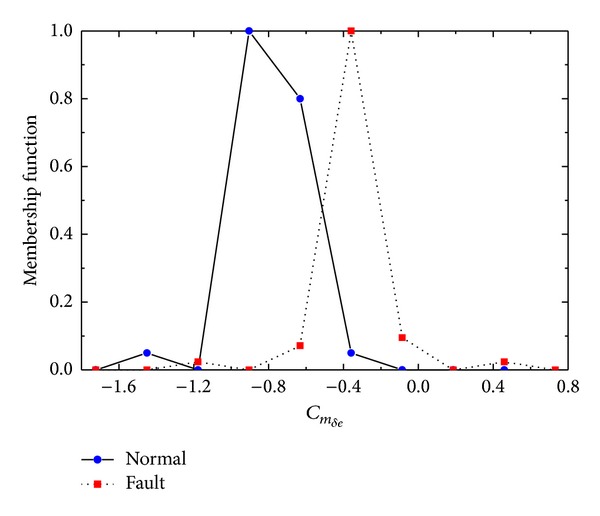
Normal/Fault data distribution of *C*_*m*_*δe*__.

**Figure 8:**
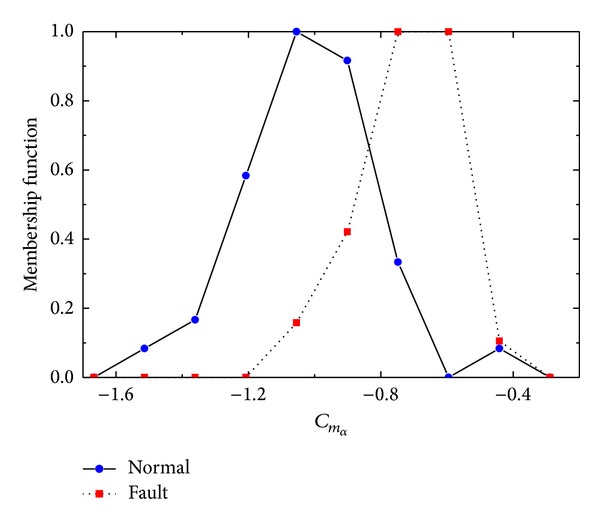
Normal/fault data distribution of *C*_*m*_*α*__.

By applying this similarity measure, calculation reduced *n* comparisons and *n* + 1 additions. Two parameter *C*_*m*_*δe*__ and *C*_*m*_*α*__ membership functions are illustrated in Figures 7 and 8. Normal and fault distributions are also shown. With similarity measure (6), decision results are clearly discriminative. In Table 5, calculation results of (6) are emphasized by considering variable importance of Table 2 as weighting factor.

## 5. Conclusions

Fault detection problem of aircraft system was carried out with RFA; it was applied to build a fault detection methodology for unmanned aircraft system, named URUMI-II. With obtained performance of RFA, results provide importance of each parameter or feature. The feasibility of fault detection algorithm with RFA was validated.

With experimental data on the flight test of DURUMI-II, fault decision algorithm showed the approach is able to detect faults with high detection rates (Figure 5). Additionally, the visualization of normal and fault patterns using MDS was able to easily figure it out with context information. Similarity measure weighting calculation with importance variable was applied for detection problem. Decision results were emphasized more than with only similarity measure.

By the calculation of RFA, meaningful result was provided that detection algorithm was effective even with a limited amount of operation data. Consequently, the flight supporting control system with fault detection algorithm is reconfigured. Then, the reliability increases without additional sensors such as a potentiometer on the control surface.

## Figures and Tables

**Table 1:** Elevator trim value in longitudinal mode (normal mode elevator trim value: 5.752).

		Elevator stuck angle				
		−10°	−5°	0°	+5°	+10°
Rudder stuck angle	−10°	—	—	−5.098	−10.008	—
	−5°	—	11.218	1.006	−9.150	—
	0°	—	10.173	5.796	−1.796	—
	5°	—	—	8.880	3.076	—
	10°	—	—	14.719	—	—

Elevator trim value (elevator stuck only)		12.640	10.173	5.796	−1.796	−9.898

**Table 2:** Variable importance with respect to feature.

Features	Variable importance
*C*_*m*_*δe*__	2.389784762
*C*_*m*_*α*__	1.601497696
*C*_*L*_*α*__	1.461898295
*C*_*D*_*δe*__	1.399035508
*C*_*D*_0__	0.939209972
*C*_*L*_*δe*__	0.734541155
$C_{m_{\dot{\alpha }}}$	0.711052447
*C*_*m*_*q*__	0.693304391
*C*_*L*_0__	0.408650397
*u*_0_	0.24089344
*C*_*D*_0__	0.196847678
*C*_*l*_*δe*__	0.121865038

**Table 3:** Polynomial coefficients and standard error.

	Value	Standard error
a	85.44917	6.34784
*b*_1_	20.15267	10.6106
*b*_2_	−11.5519	6.09265
*b*_3_	2.9505	1.61962
*b*_4_	−0.3802	0.21744
*b*_5_	0.0241	0.0143
*b*_6_	−5.97*E* − 04	3.66*E* − 04

**Table 4:** Polynomial coefficients and standard error.

	Value	Standard error
a	151.055	21.69855
*b*_1_	−142.582	54.63766
*b*_2_	143.4329	53.61102
*b*_3_	−73.217	27.62498
*b*_4_	21.30309	8.39364
*b*_5_	−3.73895	1.58132
*b*_6_	4.02*E* − 01	1.87*E* − 01
*b*_7_	−2.59*E* − 02	1.34*E* − 02
*b*_8_	9.11*E* − 04	5.38*E* − 04
*b*_9_	−1.35*E* − 05	9.19*E* − 06

**Table 5:** Computation of similarity measure.

Similarity measure		A (normal)	B (normal)	C (fault)	D (fault)
*s*_*m*_*δe*__(*F*_*N*_, *p*)		1.00	0.05	0.05	0.05
*s*_*m*_*δe*__(*p*, *F*_*F*_)		0.00	0.00	1.00	1.00
*s*_*m*_*α*__(*F*_*N*_, p)		0.33	0.08	1.00	0.92
*s*_*m*_*α*__(*p*, *F*_*F*_)		1.00	0.00	0.16	0.42

Results only (6)	s(*F*_*N*_, *p*)	**1.33**	**0.13**	1.05	0.97
	s(*p*, *F*_*F*_)	0.78	0.00	**1.16**	**1.42**

Results with Table 2	s(*F*_*N*_, *p*)	**2.92**	**0.25**	1.72	1.59
	s(*p*, *F*_*F*_)	1.60	0.00	**2.64**	**3.06**
